# *BT1549* coordinates the *in vitro* IL-10 inducing activity of *Bacteroides thetaiotaomicron*

**DOI:** 10.1128/spectrum.01669-24

**Published:** 2025-01-27

**Authors:** Morgan J. Engelhart, Orion D. Brock, Jessica M. Till, Robert W. P. Glowacki, Jason W. Cantwell, David J. Clarke, Darryl A. Wesener, Philip P. Ahern

**Affiliations:** 1 Department of Cardiovascular and Metabolic Sciences, Lerner Research Institute, Cleveland Clinic, Cleveland, Ohio, USA; 2 Department of Molecular Medicine, Cleveland Clinic Lerner College of Medicine, Case Western Reserve University, Cleveland, Ohio, USA; 3 Center for Microbiome and Human Health, Lerner Research Institute, Cleveland Clinic, Cleveland, Ohio, USA; 4 Department of Microbiology, The Ohio State University, Columbus, Ohio, USA; 5 School of Microbiology and APC Microbiome Ireland, University College Cork, Cork, Ireland; Brigham Young University, Provo, Utah, USA

**Keywords:** anti-inflammatory, gut microbiome, immune regulation

## Abstract

The intestine is home to a complex immune system that is engaged in mutualistic interactions with the microbiome that maintain intestinal homeostasis. A variety of immune-derived anti-inflammatory mediators have been uncovered and shown to be critical for maintaining these beneficial immune-microbiome relationships. Notably, the gut microbiome actively invokes the induction of anti-inflammatory pathways that limit the development of microbiome-targeted inflammatory immune responses. Despite the importance of this microbiome-driven immunomodulation, detailed knowledge of the microbial factors that promote these responses remains limited. We have previously established that the gut symbiont *Bacteroides thetaiotaomicron* stimulates the production of the anti-inflammatory cytokine IL-10 via soluble factors in a Toll-like receptor 2 (TLR2)-MyD88-dependent manner. Here, using TLR2 activity reporter cell lines, we show that the capacity of *B. thetaiotaomicron* to stimulate TLR2 activity was not critically dependent on either of the canonical heterodimeric forms of TLR2, TLR2/TLR1, or TLR2/TLR6, that typically mediate its function. Furthermore, biochemical manipulation of *B. thetaiotaomicron*-conditioned media suggests that IL-10 induction is mediated by a protease-resistant or non-proteogenic factor. We next uncovered that deletion of gene *BT1549*, a predicted secreted lipoprotein, significantly impaired the capacity of *B. thetaiotaomicron* to induce IL-10, while complementation *in trans* restored IL-10 induction, suggesting a role for *BT1549* in the immunomodulatory function of *B. thetaiotaomicron*. Collectively, these data provide molecular insight into the pathways through which *B. thetaiotaomicron* operates to promote intestinal immune tolerance and symbiosis.

**IMPORTANCE:**

Intestinal homeostasis requires the establishment of peaceful interactions between the gut microbiome and the intestinal immune system. Members of the gut microbiome, like the symbiont *Bacteroides thetaiotaomicron*, actively induce anti-inflammatory immune responses to maintain mutualistic relationships with the host. Despite the importance of such interactions, the specific microbial factors responsible remain largely unknown. Here, we show that *B. thetaiotaomicron*, which stimulates Toll-like receptor 2 (TLR2) to drive IL-10 production, can stimulate TLR2 independently of TLR1 or TLR6, the two known TLR that can form heterodimers with TLR2 to mediate TLR2-dependent responses. Furthermore, we show that IL-10 induction is likely mediated by a protease-resistant or non-proteogenic factor, and that this requires gene *BT1549*, a predicted secreted lipoprotein and peptidase. Collectively, our work provides insight into the molecular dialog through which *B. thetaiotaomicron* coordinates anti-inflammatory immune responses. This knowledge may facilitate future strategies to promote such responses for therapeutic purposes.

## INTRODUCTION

The intestinal immune system is in constant contact with microbial factors produced by the gut microbiome (1–3). Under homeostatic conditions, the gut microbiome drives the induction of anti-inflammatory immune responses that prevent unwarranted inflammatory reactions and are vital for maintaining a mutualistic relationship with the host (2, 4–6). While the microbiome imparts myriad phenotypic effects on the host immune response, the accumulation of anti-inflammatory mediators like CD4^+^ regulatory T cells and cytokines like IL-10 and TGF-β play an essential role in limiting microbiome-targeted inflammatory responses and as such are essential mediators of intestinal immune tolerance (7–17). Many members of the gut microbiome, most notably bacterial commensals, have been shown to actively induce these intestinal anti-inflammatory responses (7, 8, 10–14, 16–22), and disruption of this intricate balance precipitates the development of inflammatory intestinal disorders such as inflammatory bowel disease (23–33). Given the potent immunomodulatory capacity of the gut microbiome and the central role of this complex ecosystem in maintaining intestinal homeostasis, understanding the specific microbial mediators that drive the induction of anti-inflammatory responses is essential. However, despite huge advances in our understanding of key host immune responses that maintain host-microbiome mutualism, the microbial factors involved remain poorly defined.

Gut bacteria can shape the phenotype of the intestinal immune system through a variety of different factors, including microbially derived/modified small molecules like bile acids, short-chain fatty acids, and tryptophan metabolites, or via cell-wall components, many of which are delivered to the host via outer membrane vesicles (OMVs) (10, 16, 34–51). These cell wall products can then be sensed by pattern recognition receptors (PRRs) like Toll-like receptors (TLRs), to tailor the ensuing response based on the nature of the molecules sensed (52, 53). TLR2, has been established as a key sensor for a variety of phylogenetically distinct microbiome members (54), including several species of the *Bacteroides* genus, *Helicobacter hepaticus*, and *Bifidobacteria bifidum* (10, 17, 46, 55). TLR2 forms heterodimers with TLR1 or TLR6 that facilitate the recognition of bacterial tri- or di-acylated lipoproteins, respectively (56–59). As Gram-positive bacteria are commonly understood to make di-acylated lipoproteins while Gram-negative bacteria use an additional enzymatic step to make tri-acylated lipoproteins (60–62), it was originally hypothesized that TLR2 forms two distinct heterodimers to differentiate between Gram-positive and Gram-negative bacteria (56, 63). However, TLR2-mediated recognition of bacterial agonists has proved more complicated, with the length of the acyl-residues and even the amino acid sequence of the peptide component to which the lipid moiety is attached playing a role in their recognition and the ensuing response (64). Additionally, Gram-negative bacteria, originally thought to stimulate only TLR2/1, have also been shown to stimulate the TLR2/6 heterodimer using di-acylated glycine lipids (51, 65, 66). Moreover, while *H. hepaticus* is recognized by TLR2, neither TLR1 nor TLR6 is required, suggesting that some microbes can be equally well sensed using either heterodimer or independently of heterodimer formation (10). Thus, despite the important role of microbiome-induced anti-inflammatory responses and the prominent role of PRRs like TLR2 in mediating such responses, the microbial products that agonize these receptors to coordinate this function are poorly defined.

We previously showed that the genetically tractable gut symbiont *Bacteroides thetaiotaomicron* (*B. theta* hereafter) induces IL-10 via soluble mediators that are sensed by TLR2 (10, 17, 46, 55). Here, we sought to further investigate the specific microbial factors governing IL-10 induction by this prominent microbiome member. Our data reveal that *B. theta* produces secreted TLR2-stimulatory factor(s) that do not depend on either of the known TLRs that can form active heterodimers with TLR2 to mediate its function. Biochemical manipulation revealed that a non-proteinaceous or protease-resistant factor drove IL-10 induction and TLR2 activation. We further identified that optimal IL-10 induction was dependent on gene *BT1549*, a putative secreted peptidase and lipoprotein, while known immunomodulatory agents with predicted TLR2-stimulatory capacity, namely the CD4^+^ T cell antigen and lipoprotein encoded by *BT4295* and *glsB*-dependent glycine lipids, are dispensable for IL-10 induction. These data identify a specific gene that plays a prominent role in the capacity of the gut microbe *B. theta* to elicit anti-inflammatory immune responses, and expand our understanding of the microbial pathways that govern mutualistic interactions between the intestinal immune system and the gut microbiome.

## RESULTS

### *B. theta* produces a secreted factor sensed via TLR2 that does not critically depend on TLR2/1 or TLR2/6 heterodimers

**Fig 1:**
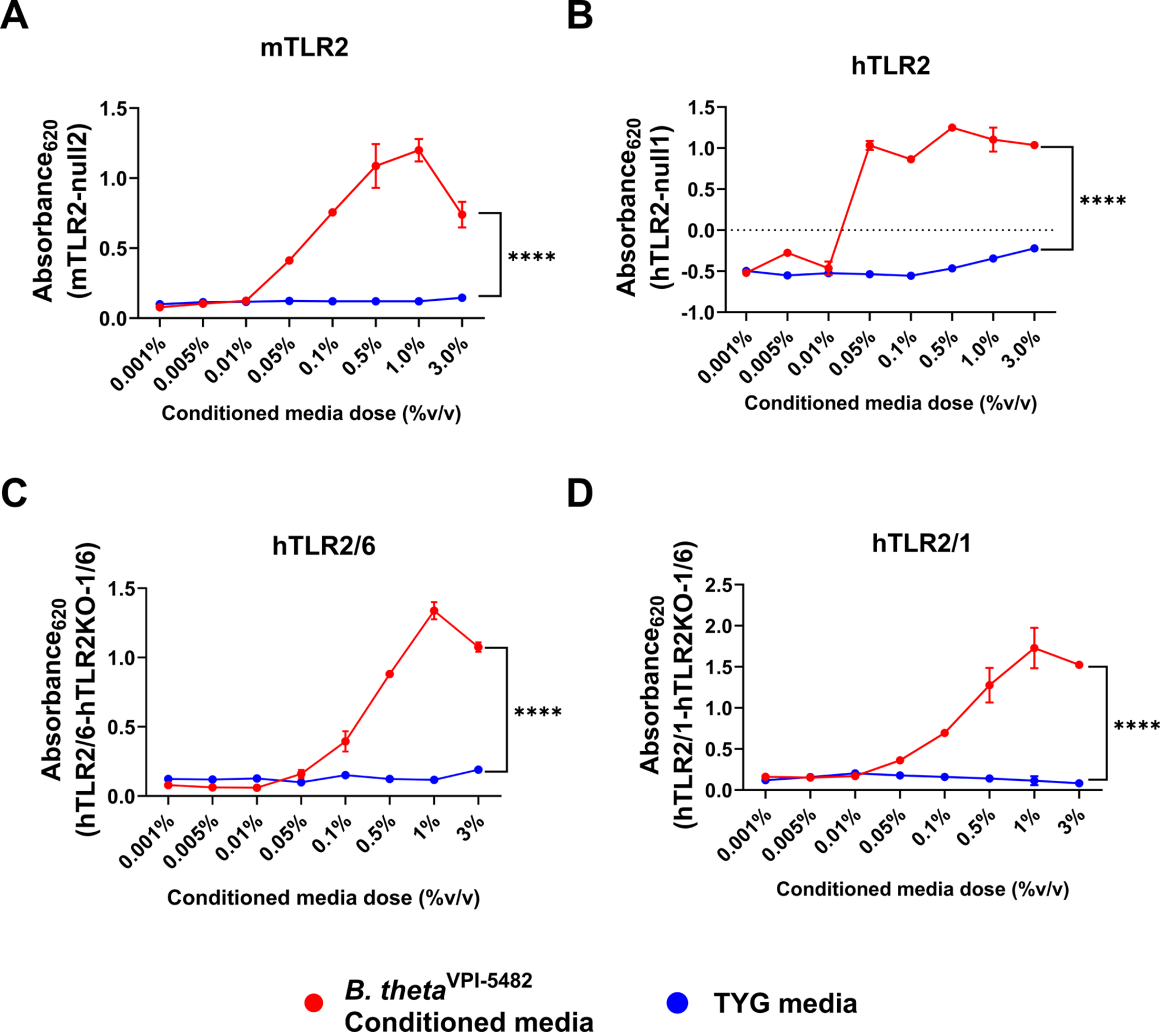
*B. theta* VPI-5482 conditioned media stimulates human and murine TLR2. (**A**) Mouse TLR2 reporter HEK293 cells or the parental cell line (null2) were stimulated with *B. theta*^VPI-5482^ conditioned media or TYG control media at the indicated doses for 20 h and TLR2 stimulation was assessed by quantification of secretion of embryonic alkaline phosphatase (SEAP)-mediated hydrolysis of detection media which can be measured by absorbance at 620 nm. Background signal was corrected by subtracting null2 absorbance values from mTLR2 absorbance values for each individual treatment group. (**B–D**) Human TLR2 (**B**), TLR2/6 (**C**), and TLR2/1 (**D**) reporter HEK293 cells, or the parental cell line, null1 for hTLR2, and TLR2KO-TLR1/6 for hTLR2/6 and hTLR2/1 cells, were stimulated with *B. theta*^VPI-5482^ conditioned media or TYG control media at the indicated doses for 20 h and TLR2 stimulation was assessed by quantification of secretion of embryonic alkaline phosphatase (SEAP)-mediated hydrolysis of detection media which can be measured by absorbance at 620 nm. Background signal was corrected for by subtracting null1 absorbance values from hTLR2 absorbance values, or hTLR2KO-TLR1/6 absorbance values from either hTLR2/1 or hTLR2/6 absorbance values for each individual treatment group. Data points represent the mean and the error bars represent the standard deviation, *n* = 2 technical replicates per dose (**A–D**). Graph is representative of three experiments (**A–D**). Statistical significance was determined using a Student’s *t* test on the area under the curve (**A–D**). ****P ≤ 0.0001.

*B. theta* has been shown to be a potent modulator of the host immune system, capable of inducing anti-inflammatory immune responses (55, 67–69). Using the induction of the anti-inflammatory cytokine IL-10 as a proxy for the anti-inflammatory capacity of *B. theta*, we have shown that it secretes immunomodulatory factors packaged within OMVs that induce IL-10 in a TLR2-dependent manner (55). Using TLR2 HEK293 reporter cells, we sought to further investigate this TLR2-dependency and characterize the *B. theta*-specific factors responsible for imprinting the anti-inflammatory properties of *B. theta*, together with IL-10 induction. HEK293 TLR2 reporter cells report NF-κB and AP-1 activity downstream of human or murine TLR2 through NF-κB/AP-1 driven release of “secretion of embryonic alkaline phosphatase” (SEAP) (70, 71), whose activity can be used as a proxy for TLR2 stimulation. To determine if *B. theta* produces a secreted factor that is recognized by both murine and human TLR2, we treated murine TLR2 (mTLR2) and human TLR2 (hTLR2) HEK293 reporter cells (and the corresponding parental cell lines) with different doses of *B. theta* (strain VPI-5482) conditioned media (sterile filtered TYG media conditioned by growth of *B. theta* to stationary phase) or TYG control media, and measured receptor activation via SEAP production. As expected, *B. theta* conditioned media significantly activates both murine and human TLR2 compared to TYG control media (Fig. 1A and B), in keeping with the role of TLR2 in mediating responsiveness to *B. theta* (55). TLR2 senses di-acylated agonists via a TLR2/TLR6 heterodimer, and tri-acylated agonists via a TLR2/TLR1 heterodimer (56, 60–63). However, additional biochemical features of agonists modulate TLR2 activation including acyl-chain length and amino acid sequence (64). We treated hTLR2/1 HEK293 reporter cells (cells are co-transfected with TLR2 and TLR1 and do not contain TLR6) and hTLR2/6 HEK293 reporter cells (cells are co-transfected with TLR2 and TLR6 and do not contain TLR1) (and the corresponding parental cell line), with different doses of *B. theta* conditioned media or TYG control media and measured receptor stimulation, to investigate whether *B. theta* produced secreted factors capable of activating TLR2/1 or TLR2/6 heterodimers. To our surprise, *B. theta* conditioned media significantly activated both hTLR2/1 and hTLR2/6 reporter cells compared to TYG control (Fig. 1C and D), thus suggesting that *B. theta* produces secreted factors that can be recognized independently of TLR1 (TLR2 stimulation is observed in the TLR2/TLR6 reporter which lacks TLR1) and independently of TLR6 (TLR2 stimulation is observed in the TLR2/TLR1 reporter which lacks TLR6). These data suggest that there may be redundancy in the TLR2-mediated recognition of *B. theta* with either TLR1 or TLR6 able to mediate responsiveness. However, despite the fact that Pam3CSK4, a tri-acylated lipopeptide, is expected to only stimulate TLR2 via a TLR2/TLR1 heterodimer, we found that it induced activation of hTLR2/6 cells at the highest doses tested (5 and 10 ng/mL), in keeping with reports suggesting a dose dependency in the requirement for TLR1 and results obtained by the generator of the cell line (personal communication, Invivogen) (Fig. S1) (64). By contrast, Pam2CSK4 showed a strict dependency on TLR2/TLR6, as even at high doses, it did not appreciably stimulate TLR2/TLR1 reporter cells (Fig. S1). Importantly, although responsiveness to *B. fragilis* operates via a TLR2/TLR1 axis (72), other gut microbes that are sensed via TLR2 can promote IL-10 production even when TLR1 or TLR6 is absent (10). These data suggest that *B. theta* produces secreted factors capable of activating TLR2 via either a TLR2/TLR1 or a TLR2/TLR6 combination, independently of both heterodimeric forms, or potentially via a TLR2 homodimer.

**Fig 2:**
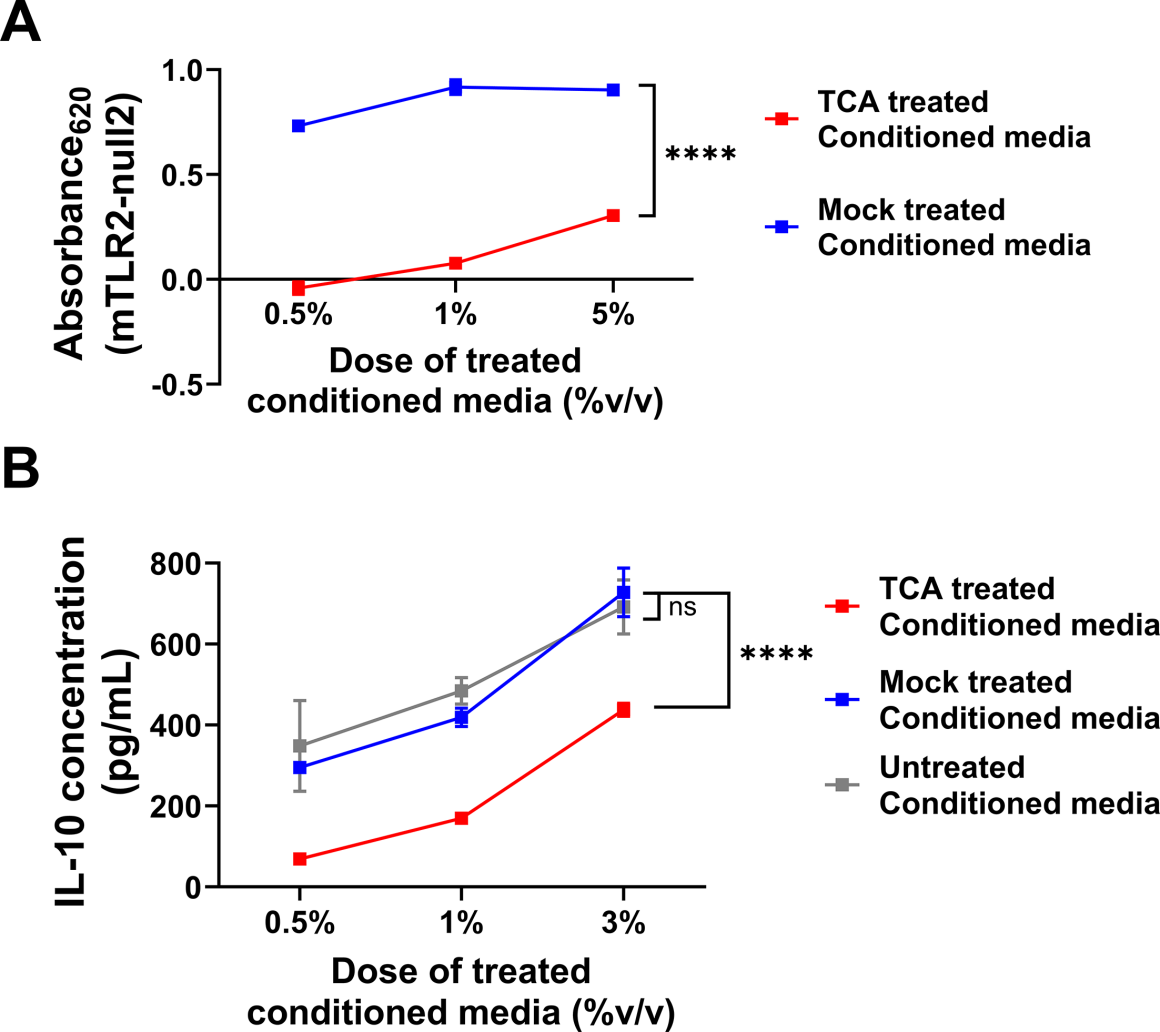
*B. theta* VPI-5482 produces a TCA-precipitable factor that stimulates TLR2 and induces production of IL-10. (**A**) Mouse TLR2 reporter HEK293 cells and parental cell line (null2) were stimulated with *B. theta*^VPI-5482^ dialyzed or TCA protein-depleted conditioned media at the indicated doses for 20 h and TLR2 activation was assessed by quantification of secretion of embryonic alkaline phosphatase (SEAP)-mediated hydrolysis of detection media which can be measured by absorbance at 620 nm. Background signal was corrected for by subtracting null2 absorbance values from mTLR2 absorbance values for each individual treatment group. (**B**) Wild-type splenocytes were stimulated with *B. theta*^VPI-5482^ TCA treated conditioned media, mock-treated or -untreated conditioned media controls at the indicated doses for 2 days and IL-10 was assessed in the supernatant by ELISA. Data points represent the mean and the error bars represent the standard deviation, *n* = 2 technical replicates per dose (**A and B**). Graph is representative of three independent experiments (**A and B**). Statistical significance was determined using a Student’s *t* test (**A**), or one-way ANOVA with a Dunnett’s *post hoc* test and comparisons to the mock-treated conditioned media (**B**) on the area under the curve. ^ns^*P* ≥ 0.05, ****P ≤ 0.0001.

To further investigate the IL-10 inducing factor produced by *B. theta*, we aimed to biochemically characterize the agonist(s) found within the conditioned media. TLR2 recognizes lipid moieties bound to proteins and potentially carbohydrates (10, 49, 56, 73). To determine whether the active factor is bound to a protein, we performed trichloroacetic acid (TCA)-based protein precipitation (74) of *B. theta* conditioned media followed by extensive dialysis and found that protein depletion diminished the ability of *B. theta* conditioned media to activate TLR2 and to induce IL-10 compared to mock precipitated controls (Fig. 2A and B). Importantly, spiking Pam3CSK4 into wells treated with TCA-treated or mock-treated control conditioned media readily drove high levels of IL-10, suggesting a loss/reduction in the factor necessary for induction of IL-10 due to the TCA treatment rather than residual TCA inhibiting cell responses (Fig. S2A and B). TCA treatment lowers pH, and thus to determine if the impact of TCA could be truly ascribed to protein precipitation of the active factor as opposed to destruction of the factor, we used two orthogonal methods of protein depletion, methanol-based or acetone-based protein precipitation, and found that, as with TCA-treatment, both treatment types lead to a significant loss of TLR2 activity and reduced capacity to stimulate the production of IL-10 (Fig. S3A through C). Notably, TLR2-stimulatory activity and IL-10 inducing capacity could be recovered in the precipitated pelleted material of all three treatment types, further supporting the notion that the TLR2 agonist was being precipitated rather than being damaged (Fig. S3A through C). However, despite their known capacity to precipitate proteins, we cannot exclude that these precipitation methods are not also precipitating non-protein factors such as polysaccharides or other glycoconjugates which have previously been linked to TLR2-dependent stimulation of IL-10 via attached lipid moieties.

Given the prominent role of lipoproteins in TLR2 stimulation coupled with the fact that the removal of proteinaceous material via precipitation led to reduced TLR2 stimulation and IL-10 induction, we reasoned that *B. theta* produces a lipoprotein (a protein with an attached lipid moiety) that was responsible for TLR2 activation and IL-10 induction. However, digestion of proteinaceous material within the conditioned media with proteinase K or pronase (75, 76) only modestly reduced TLR2 stimulation compared to dialyzed conditioned media controls (the reduction in TLR2 stimulation was not equivalent to that seen with TCA precipitation) (Fig. S4A), and did not hinder the ability of *B. theta* conditioned media to induce IL-10 from wild-type splenocytes compared to dialyzed conditioned media or untreated conditioned media controls (Fig. S4B). These data suggest that the various precipitation methods may also have precipitated non-proteinaceous factors. Moreover, the fact that TLR2 stimulation was reduced but IL-10 induction was unimpaired suggests that once a certain level of TLR2 stimulation is achieved, further stimulation cannot enhance IL-10 production any further in this assay system.

As with TCA treatment, Pam3CSK4 supplemented to either protease-treated sample after treatment drove equivalent activity irrespective of treatment type (Fig. S2C) showing that treatment with these proteases did not impair assay performance. Given the surprising discrepancy in IL-10 induction between the two approaches used to deplete proteins from the conditioned media, we sought to determine the efficacy of both methods. Silver-staining of SDS-PAGE gels loaded with the treated and control conditioned media revealed that both the precipitation and digestion-based approaches led to efficient removal of the proteinaceous material in the conditioned media, albeit with the presence of potentially protease-resistant bands notable in the proteinase K and pronase-treated samples at roughly 17 and 10 kDa (Fig. S5A and B). Emerald staining, which brightly labels carbohydrates (present as polysaccharides or glycoconjugates) after diol oxidation, suggests these protease-resistant bands (plus high molecular weight bands that do not enter the gel) comprised or contain carbohydrates (Fig. S5C). Importantly, following a liquid-liquid extraction of the conditioned media, the agonist(s) is readily extracted into the aqueous phase (Fig. S5D). Therefore, even though it is expected that a lipid moiety is required for TLR2 stimulation, the data suggest that free lipids are not responsible. As such, the protease-resistant bands, which we propose to contain carbohydrates, may have a lipid moiety attached that contributes to stimulate TLR2 and induce IL-10 (56, 64, 73). Although our data point to a non-proteinaceous factor, we cannot exclude the scenario that proteinase K and pronase leave lipidated peptides sufficiently intact such that they were not removed by dialysis, and can activate the TLR2-IL-10 axis.

### Gene *BT1549* encodes a predicted lipoprotein required for *B. theta*-mediated IL-10 induction

We next sought to identify the specific pathways in *B. theta* that were responsible for IL-10 induction. Genes *BT3459* and *BT4295* have been previously implicated in the immunomodulatory function of *B. theta* (77, 78). Gene *BT3459* (*B. theta*^Δ*tdk*Δ*BT3459*^; *BT3459* referred to as *glsB* hereafter) encodes an N-acyltransferase activity required for the first step in the generation of glycine lipids, known TLR2 agonists (79). Therefore, *glsB* deficient *B. theta* cannot produce glycine lipids. However, we observed that *glsB*-deficient *B. theta (B. theta*^Δ*tdk*Δ*BT3459*^) induced equivalent levels of IL-10 as the parental strain control *B. theta (B. theta*^Δ*tdk*^; the absence of *tdk* facilitates counter selection during mutagenesis), or *glsB*-complemented *B. theta (B. theta*^Δ*tdk*Δ*BT3459*::*BT3459*^) (Fig. S6A) despite a slight reduction in growth (Fig. S7A and 8A). Gene *BT4295* encodes an immunodominant lipoprotein, recognized by CD4^+^ T cells, and *BT4295*-specific CD4^+^ T cells differentiate into Tregs upon antigen encounter in the intestine (80). However, we observed no reduction in the IL-10 inducing capacity of conditioned media from a *BT4295*-deficient *B. theta* mutant (*B. theta*^Δ*tdk*Δ*BT4295*^) relative to the parent strain (*B. theta*^Δ*tdk*^) (Fig. S6B). As *BT4295* expression is sensitive to media composition, we confirmed its expression under our culture conditions by showing that *B. theta*^Δ*tdk*^ but not *B. theta*^Δ*tdk*Δ*BT4295*^ could activate CD4^+^ T cells specific for this antigen (Fig. S6C and D), and additionally, observed no significant differences in growth between the mutant and parent strain (Fig. S7B and 8B). Thus, neither of these genes played an important role in *B. theta*-mediated IL-10 induction in the contexts we have tested, despite their immunomodulatory potential.

**Fig 3:**
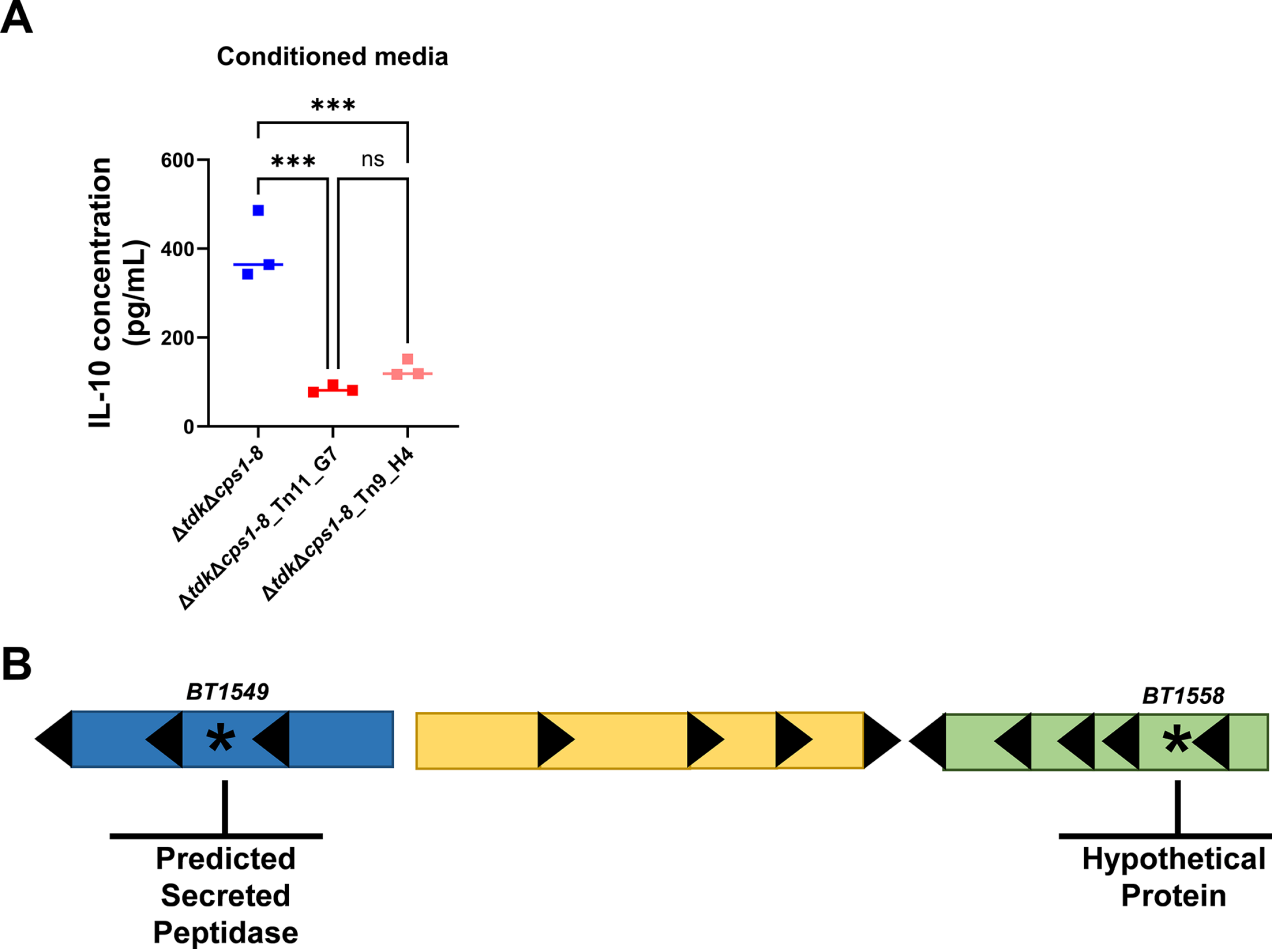
*B. theta* transposon mutagenesis library identified candidate genes required for IL-10 induction. (**A**) Wild-type splenocytes were stimulated with conditioned media (1% vol/vol) from *B. theta*^*ΔtdkΔcps1-8*^ or transposon mutants 11_G7 or 9_H4 (generated on the *B. theta*^*ΔtdkΔcps1-8*^ background) for 2 days and IL-10 was assessed in the supernatant by ELISA. (**B**) The transposon insertion in transposon mutants 11_G7 and 9_H4 mapped within a close genomic region and hit genes *BT1549* and *BT1558*. Data points represent individual technical replicates while the horizontal line represents the median, *n* = 3 technical replicates per dose (**A**). Graph is representative of four experiments (**A**). Statistical significance was determined by a one-way ANOVA with Tukey’s *post hoc* test and comparisons between all groups (**A**). ^ns^*P* ≥ 0.05 and ***P ≤ 0.001.

To further investigate the potential TLR2 agonists produced by *B. theta* that could induce IL-10 we took a less biased approach to identify genes required for this process. We previously published a *B. theta* transposon mutagenesis library screen (55) on the acapsular *B. theta*^Δ*tdk*Δ^*^cps1-8^* background that identified transposon mutants with impaired ability to induce IL-10 (55, 80) (while the capsule of many microbiome members has shown to play an important role in immunomodulation (10, 40, 45, 49, 72), we utilized a transposon mutant library on the acapsular *B. theta* background because of our interest in identifying capsule-independent mechanisms of immune-modulation). In our original screen, which identified a role for a gene that encodes a component of the NQR complex in IL-10 induction (55), we identified several additional transposon mutants of unknown transposon insertion location with decreased ability to induce IL-10. We first sought to validate these hits from the original screen by generating an independent batch of conditioned media using these mutants, and confirmed that their ability to induce IL-10 was impaired compared to the parent strain (*B. theta*^Δ*tdkΔcps1-8*^; note, mutants are named for the multi-well plate from which they were obtained, followed by the well in which the mutant resides, e.g., mutant 11_G7 is derived from plate 11 and well G7) (Fig. 3A).

Interestingly, when we mapped the genomic location of transposon insertion within *B. theta*, we found that the transposon for each mutant had been inserted into genes relatively close together in the genome, within two distinct bacterial operons only separated by a few genes (Fig. 3B). Transposon mutant 11_G7 contained an insertion in gene *BT1549*, which is a predicted secreted peptidase, and transposon mutant 9_H4 had an insertion in gene *BT1558*, a hypothetical protein belonging to the beta-barrel outer membrane protein family (81) (Fig. 3B). Given the link between lipoproteins and TLR2, we interrogated these genes using the server SignalP-6.0 (https://services.healthtech.dtu.dk/services/SignalP-6.0/; fast mode settings) which can be used to predict the presence of lipoprotein signal peptides using the amino acid sequence encoded by each gene (82). Using SignalP-6.0, we identified that the protein encoded by gene *BT1549* is a predicted lipoprotein due to the high probability of a Sec/SPII signal peptide sequence (Fig. S9A and B; predicted probability of 0.8974) (it is important to note that this is a predicted feature and confirmation of this requires biochemical verification). However, gene *BT1558* did not contain any signal peptide and therefore is not predicted to be a secreted protein or a lipoprotein (data not shown). Of note, both genes encode proteins packaged within *B. theta* OMVs (83), and *B. theta*-derived OMVs have been shown to interact with and modulate the immune system (55, 84).

**Fig 4:**
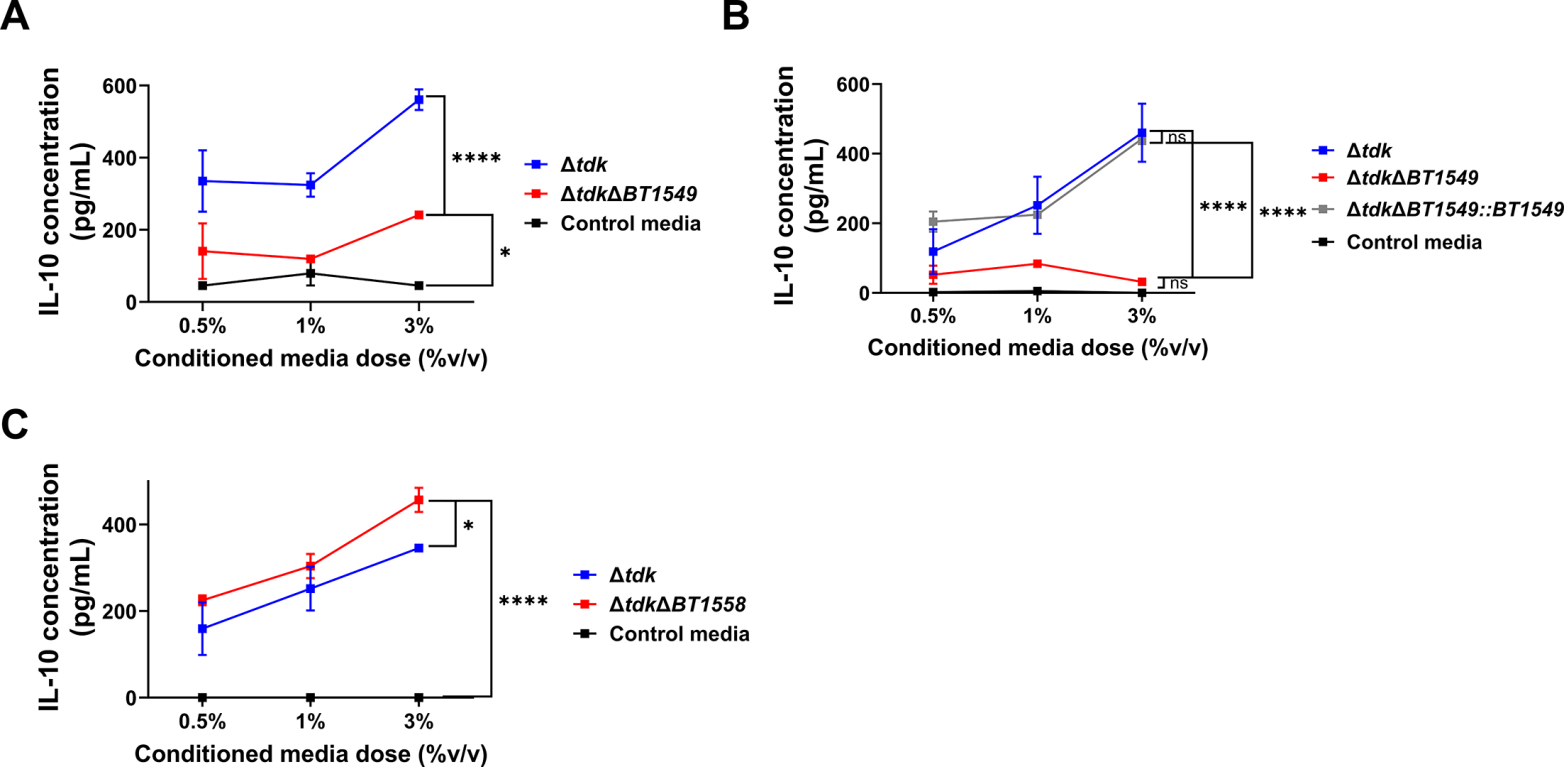
Gene *BT1549*, a predicted lipoprotein, but not gene *BT1558* is required for *B. theta*-mediated IL-10 induction. (**A–C**) Wild-type splenocytes were stimulated with conditioned media at the indicated doses from *B. theta*^*Δtdk*^ or *B. theta* harboring a deletion in gene *BT1549* (*B. theta*^*ΔtdkΔ1549*^) (**A**) or *B. theta*^*Δtdk*^, *B. theta*^*ΔtdkΔBT1549*^, or *B. theta*^*ΔtdkΔBT1549::1549*^ (**B**) or *BT1558* (*B. theta*^*ΔtdkΔt1558*^) (**C**) or TYG control media for 2 days and IL-10 was assessed in the supernatant by ELISA. Data points represent the mean and error bars represent the standard deviation, *n* = 2 technical replicates per dose (**A–C**). Graph is representative of three experiments (**A and C**) and two experiments (**B**). Statistical significance was determined using a one-way ANOVA with a Dunnett’s *post hoc* test and comparisons to the mutant of interest (**A and B**) or Tukey’s *post hoc* test and comparisons between all groups (**C**) on the area under the curve. ^ns^*P* ≥ 0.05, *P < 0.05, ****P ≤ 0.0001.

To validate the role of these genes in the ability of *B. theta* to induce IL-10 we generated full, singular deletions of either gene, *BT1549* or *BT1558*, on a capsular sufficient genetic background (*B. theta*^*Δtdk*^). Deletion of gene *BT1549* (*B. theta*^Δ*tdkΔBT1549*^) resulted in a significant impairment of *B. theta* conditioned media to induce IL-10 compared to the parent strain (*B. theta*^Δ*tdk*^) (Fig. 4A; Fig. S8C), and was not likely a result of differences in the growth between mutant and the parent strain (Fig. S7C). Complementation of *BT1549 in trans* by introduction of the full-length gene and predicted promoter region into *att2* (tRNA^ser^) in *B. theta*^Δ*tdkΔBT1549*^ (*B. theta*^Δ*tdkΔBT1549::BT1549*^) restored the ability of *B. theta* to induce IL-10, back to the level of the parent strain (Fig. 4B; Fig. S8C), suggesting a role for this gene in IL-10 induction. Deletion of gene *BT1558*, however, did not recapitulate the findings with the transposon mutant and showed no impairment in IL-10 induction compared to the parent strain (Fig. 4C), suggesting that the impact of the transposon insertion may be through modulation of downstream genes. These data suggest that gene *BT1549* is required for *B. theta* to induce IL-10, but that *BT1558* does not play a prominent role in this process. As we have previously established that OMVs play an important role in *B. theta*-mediated IL-10 induction (55), we assessed the impact of deletion of *BT1549* on OMV biogenesis. However, we found that the deletion of gene *BT1549* did not impact OMV production (Fig. S9C), and thus, the effects of *BT1549* are mediated by a distinct mechanism.

## DISCUSSION

The dynamic relationship between the intestinal immune system and the gut microbiome has fascinated and bewildered scientists for decades. While much of the early work investigating the relationships between the immune system and microbes has focused on pathogen recognition and response, more recently, the important role the gut microbiome plays in maintaining intestinal homeostasis by induction of anti-inflammatory responses has led to an explosion of interest in understanding the mechanistic underpinnings of these responses given their potential utility to prevent diseases.

The recognition of microbe-associated molecular pattern molecules via PRRs represents a primary mechanism through which the immune system senses bacteria and mounts the appropriate quality of immune response. Several studies have shown that engagement of host PRRs by gut microbiome-derived factors like cell wall products, helps to maintain intestinal homeostasis by inducing anti-inflammatory immune responses and normative tissue development (2, 4–6, 85). One such PRR, TLR2, mediates responsiveness to many microbes that exert anti-inflammatory effects (10, 17, 55, 72). Stimulation of the TLR2 axis to induce anti-inflammatory immune responses is thus emerging as a common process through which microbiome members can promote immune tolerance. We previously established that the gut symbiont *B. theta* is also recognized via TLR2 (55). TLR2 recognizes a broad array of agonists by forming heterodimers with TLR1 and TLR6 to recognize tri- or di-acylated lipoproteins and glycine lipids (56, 60–63, 65, 66), and thus understanding which heterodimeric form of TLR2 is stimulated can provide insights into the biochemical features of the agonists. Our data reveal that *B. theta* produces secreted factors that do not critically depend on TLR1 or TLR6. Although not in keeping with a model where a given microbe can only interact with one of these heterodimers as in the case of *B. fragilis* (45), these data are consistent with work showing that *H. hepaticus* stimulates IL-10 through TLR2, but independently of TLR1 and TLR6 (10). Thus, both TLR2/TLR1 and TLR2/TLR6 may collectively sense products of gut microbiome members, or it remains possible that neither TLR1 or TLR6 is required and signals may instead be mediated by homodimeric forms of TLR2 (64). Glycine lipids, di-acylated amino acid lipids, have been shown to activate TLR2/6 and exert anti-inflammatory responses (51, 65, 66, 86) and are produced by *B. theta* in a *glsB*-dependent manner (79). Despite the clear role of these lipids in TLR2 activation, our data suggest that *glsB*-dependent glycine lipids are not required for IL-10 induction by *B. theta* as a mutant lacking the ability to produce any glycine lipids showed no impairment in stimulating IL-10 production. Thus, while *glsB*-dependent glycine lipids may contribute to the anti-inflammatory function of *B. theta* they are not absolutely required to induce such responses.

Numerous studies have identified *B. theta*-specific genes required for its immunomodulatory capacity and colonization fitness (48, 55, 79, 80). Our data now provide evidence of another *B. theta* gene that plays a key role in its immunomodulatory function and is intricately involved in its ability to induce IL-10 via TLR2. We identified gene *BT1549*, a predicted lipoprotein and peptidase, as required for *B. theta* to induce IL-10, and showed that complementation of *BT1549 in trans* could restore near wild-type levels of IL-10 induction, suggesting a role for this gene in inducing this response. However, we found that deletion of another known immunomodulatory factor that is also a predicted lipoprotein (encoded by gene *BT4295*) (80) did not impair its ability to induce IL-10. Moreover, inhibition of glycine lipids, di-acylated amino acids known to agonize the TLR2 axis (51, 65, 66, 86), through deletion of *glsB* (79) also did not impair IL-10 induction. Thus, while *BT4295* and *glsB*-dependent glycine lipids may contribute to the anti-inflammatory function of *B. theta* they are not absolutely required to induce such responses. Interestingly, the predicted lipoprotein encoded by *BT4295* is exquisitely sensitive to dietary components for its expression (80), and thus, it is possible that the precise factors that drive induction of anti-inflammatory cytokines may depend on other environmental factors rather than being limited to a given agent. Such redundancy would ensure that alteration in the expression of a given factor would not impair immunomodulatory capacity. One strategy to test if there is a role more broadly for lipoproteins in the response is the deletion of the LGT (Phosphatidylglycerol::Prolipoprotein Diacylglycerl Transferase) enzyme, which catalyzes the first step in lipoprotein formation (60, 61). However, we were unable to generate a deletion mutant of this gene in *B. theta* in keeping with results in other Gram-negative bacteria where the gene appears essential to the bacterium’s survival (87, 88), by contrast with Gram-positive bacteria.

Our finding that protein depletion from conditioned media reduced IL-10 induction implicated proteinaceous material in driving this response, in keeping with a role for lipoproteins like *BT1549* in this response. However, the failure of proteinase K or pronase treatment to deplete IL-10 induction capacity by *B. theta* conditioned media, despite efficient protein degradation, suggests that a non-protein factor may be involved. Notably, a protease-resistant band was evident implicating a factor recalcitrant to digestion/complete digestion, and this band was evident on Emerald-stained gels, suggesting the presence of carbohydrates/glycoconjugates. Thus, it is possible that carbohydrates could also have been precipitated by different precipitation methods, in addition to protein, and is in keeping with the role of cellular polysaccharides in mediating the anti-inflammatory effects of various gut bacteria. Indeed, mounting evidence supports a role for cellular polysaccharides produced by gut bacteria like *B. fragilis*, *B. bifidum*, and *H. hepaticus* that can promote IL-10 production via TLR2, providing a strong precedent for a microbiome/TLR2/IL-10 axis in response to polysaccharides (10, 17, 40, 45). Despite this, challenges associated with the purification of bacterial cellular components to homogeneity mean that an unidentified component that co-purifies with carbohydrates could be responsible for the IL-10 inducing activity of particular carbohydrate preparations (89). Indeed, when polysaccharide A (PSA) from *B. fragilis*, a potent inducer of IL-10, was chemically synthesized, it was found to lack the activity of PSA purified from cells (72). This difference was linked to the presence of the lipid anchor of the capsular polysaccharide, which is found in purified but not in synthesized PSA. Furthermore, lipoproteins embedded within peptidoglycan have also led to the peptidoglycan being inadvertently identified as a TLR2 activating agent (89). Given the prominence of lipid-recognition in the stimulation of TLR2 (56), it is evident that much work is required to further define the active components involved. Importantly, *BT1549* is predicted to be one of many peptidases encoded by the *B. theta* genome (90, 91). Microbial peptidases are a broad class of enzymes that have been shown to play a role in the gastrointestinal tract in host nutrient utilization and degradation of the host extracellular matrix by degradation of both dietary and host-derived proteins (92) and as such, the peptidase activity could be required for the induction of IL-10, although it is important to note that this function is a prediction and to the best of our knowledge peptidase activity has yet to be biochemically established for this protein. Ultimately, although our data do not suggest that *BT1549* mediates its role directly, the fact that complementation *in trans* restored IL-10 induction to near wild-type levels suggests that the effect of deletion of *BT1549* is unlikely to be due to effects on nearby genes.

Collectively, our studies have identified a novel (to our knowledge) pathway required for the ability of *B. theta* to induce IL-10 via TLR2. The ability to modulate anti-inflammatory mediators by targeting the gut microbiome remains an attractive therapeutic goal. Identifying a specific gene required for the ability of *B. theta* to induce IL-10 provides a promising target to be used in generating probiotics to treat inflammatory diseases such as inflammatory bowel disease. However, further studies are required to purify and identify the TLR2 agonist whose abundance/activity is impacted by gene *BT1549*. Identification of the specific molecular factors mediating the effects of distinct microbiome members will shed light on whether TLR2 responds to a variety of microbiome-derived agonists to promote anti-inflammatory immune responses, or if there is a single molecular species common to a large array of such microbes that intersect with the TLR2 pathway to promote immune tolerance. Moreover, the identification of such agonists may facilitate the development of adjuvants designed to promote anti-inflammatory immune responses for therapy. Finally, defining the cell types that respond to microbiome-derived agonists and act as sources of anti-inflammatory mediators will shed light on how mutualistic interactions are maintained within the intestine. While our previous studies suggest macrophages can produce TLR2-dependent IL-10 in response to *B. theta in vitro* (55), agreeing with other studies that show a role for macrophages in producing IL-10 in response to gut microbes (10), several other cell types may be involved. Specifically, Tregs may act as important sources of anti-inflammatory cytokines that reinforce a cytokine and cellular milieu that favors immune tolerance.

## MATERIALS AND METHODS

### Animals

Spleens and bone marrow used were isolated from C57BL/6J animals (The Jackson Laboratory, Stock #000664) or BθOM TCR transgenic animals on a C57BL/6J RAG1^−/−^CD45.1^+^CD45.2^−^ background (77), whose use was approved by the Institutional Animal Care and Use Committee (IACUC) of the Cleveland Clinic.

### Bacterial strains, culturing conditions, and molecular genetics

*B. thetaiotaomicron* ATCC 29148 (VPI-5482) and its genetic variants were grown in tryptone-yeast extract-glucose (TYG) broth medium (55) at 37°C in an anaerobic chamber (compressed gas: 5% CO_2_, 5% H_2_, 90% nitrogen, chamber set to ~2.5% H_2_, Coy Manufacturing, Grass Lake, MI, USA). Genetic deletions of target genes were performed using the established counter-selectable allelic exchange approach following previously described procedures (55, 93). All bacterial strains, plasmids, and primers used are described in Table S1A. In brief, deletion of gene *BT1549* or *BT1558* was done using a modified form of *B. theta* strain VPI-5482 harboring a deletion of the thymidine kinase gene, *tdk* (*BT2275*), to facilitate counter-selection. Genes were deleted by ligating PCR-amplified 750 bp fragments flanking the gene of interest into the suicide pExchange-*tdk* vector using Sal1 (NEB, Cat. #R0138L) and Xbal (NEB, Cat. # R0145L) cut sites. pExchange-*tdk* contains a cloned copy of *tdk* (provides susceptibility to the toxic nucleotide analog 5-fluoro-2′-deoxyuridine [FUdR]), *bla* (ampicillin resistance), and *ermGb* (erythromycin resistance), to facilitate selection and counter-selection. The resulting construct was conjugated into either *B. theta*^*Δtdk*^ or *B. theta*^*ΔtdkΔcps*1-8^. Individual-recombinant merodiploid colonies, selected on erythromycin (25 μg/mL), were pooled and plated on BHI-blood agar containing FUdR (200 μg/mL) to select for recombinants. Candidate gene deletions were screened by PCR and DNA sequencing to identify isolates that had the desired gene deletions.

Gene *BT1549* complementation *in trans* was accomplished as previously described (55, 94). In brief, the wild-type *BT1549* and predicted promoter region were PCR-amplified using the primers described in Table S1A. Complementing alleles were ligated into the *pNBU2-ermGb* vector using Sal1 and BamH1 (NEB, Cat. #R0136L) cut sites and the resulting construct was inserted into *att2* (tRNA^ser^) in *B. theta*^*ΔtdkΔBT1549*^. Individual recombinant colonies selected on erythromycin (25 μg/mL) were screened by PCR and DNA sequencing to identify successfully complemented isolates.

### *B. theta* conditioned media

The indicated *B. theta* strains/mutants were grown overnight under anaerobic conditions in TYG growth medium to stationary phase (~16 h) and the OD_600_ measured (ranging from ~2.0 to 3.5). Conditioned media were generated by normalizing the OD_600_ of bacterial cultures being directly compared using TYG, then pelleted by centrifugation for 5 min at 7000 × *g* at 4°C, and the supernatant was filtered through a 0.22 μm PVDF filter (Millex, Cat. #SLGVR33RS) to make aliquots of cell-free sterile supernatant (conditioned media) that was frozen at −20°C until further use. The effectiveness of sterile filtering the conditioned media was confirmed by plating on BHI blood agar plates and incubating at 37°C anaerobically for >3 days (data not shown).

### *In vitro* IL-10 assay

The ability of *B. theta* conditioned media from the indicated strains or mutants to induce IL-10 was tested by culturing with bulk unfractionated murine splenocytes *in vitro* (55). In brief, splenocytes were isolated from the spleen of wild-type C57BL/6 mice to generate a single-cell suspension in sterile PBS containing 0.1% wt/vol bovine serum albumin (BSA; Sigma). To lyse red blood cells, the spleen pellets were incubated in ACK lysis buffer (Gibco, Cat. #A10492) for 3 min after which ~9 mL of PBS/BSA was added, and the cell suspension was then passed through a 70 μm cell-strainer into a new 15 mL tube. Complete RPMI (RPMI 1640 supplemented with l-glutamine, 20 mM final concentration HEPES (Gibco, Cat #15630-080), 10% vol/vol final concentration heat-inactivated fetal bovine serum (FBS), 100 units/mL Penicillin and 100 μg/mL Streptomycin were used to resuspend spleen cells and live cells were counted by hemocytometer (stained with trypan blue) and diluted with complete RPMI to 5 × 10^6^ cells/mL. Splenocytes were plated at 5 × 10^5^ cells/well in a flat-bottomed sterile 96-well tissue culture-treated plate (Gibco, Cat. #353072) together with the indicated doses of conditioned media, and additional complete RPMI was added to bring the final well volume to 200 μL. The cultures were incubated at 37°C in 5% CO_2_ for 48 h, following which the cells were pelleted by centrifugation at 454 × *g* for 5 min and the supernatants were collected. Supernatants were frozen at −20°C until further processing, and IL-10 was measured by ELISA (BioLegend, Cat. #431411).

### *In vitro* TLR2 reporter assay

The ability of *B. theta* conditioned media and *B. theta*-treated conditioned media to activate TLR2 was tested using TLR2 reporter HEK293 cells. Conditioned media were tested on HEK293 cells expressing (i) murine TLR2 (Invivogen, Cat #hkb-mtlr2), (ii) human TLR2 (Invivogen, Cat #hkb-htlr2), and (iii) human TLR2/1 (Invivogen, Cat #hkb-htlr21), and human TLR2/6 (Invivogen, Cat #hkb-tlr26) reporter cells. Parental cell lines null1 (Invivogen, Cat #hkb-null1), null2 (Invivogen, Cat #hkb-null2), and the control cell line for hTLR2/1 and hTLR2/6 reporter lines, human TLR2KO-TLR1/6 (Invivogen, Cat #hkb-htlr2k16; cells are deficient in TLR1 and TLR6) were also used. The cells were thawed and handled according to the manufacturer’s instructions. Briefly, the cells were thawed at 37°C in a water bath by gently shaking the vial. The vial was removed from the water bath while a small frozen cell pellet remained. The outside of the vial was cleaned with 70% ethanol again. Cells were transferred to a 50 mL conical tube containing 15 mL prewarmed complete DMEM (l-glutamine and Na Pyruvate, supplemented with HEPES (20 mM final concentration; Gibco, Cat #15630-080), 10% vol/vol final concentration heat-inactivated FBS, 100 units/mL Penicillin, and 100 μg/mL Streptomycin). The cells were spun down at 454 × *g* for 10 min at room temperature and the supernatant was carefully removed. The cells were resuspended in 5 mL complete DMEM and cultured in a T25 tissue culture-treated flask (Corning, Cat #430639). Media were changed twice a week and cells were split, expanded, or used when they reached ~70% confluency. After two passages, the cells were split and given new complete DMEM containing specific selection antibiotics (Invivogen, Zeocin [Cat #ant-zn-05], Normocin [Cat #ant-nr-05], and HEK-BlueTM Selection [Cat #hb-sel]).

The cells were harvested into sterile PBS by gently tapping the flask when they reached ~70% confluency. The cells were counted using a hemocytometer (stained with trypan blue) and diluted to 2.8 × 10^5^ cells/mL with HEK-Blue Detection media (powder dissolved in sterile water, according to the manufacturer’s instructions, and sterile filtered). Prior to cells being harvested, *B. theta* conditioned media samples were diluted to 10× the desired concentration and 20 μL was plated per well in a flat-bottomed sterile 96-well tissue culture-treated plate (Gibco, Cat #353072). About 180 μL of HEK293 cells diluted to the desired concentration using Detection media was added to each well. The cells were incubated for ~20 h at 37°C in 5% CO_2_. SEAP-activation (corresponding to the appropriate TLR2 activation depending on the cell line) was measured using absorbance at 620 nm. Any background signal for mTLR2 HEK cells was corrected using the parental cell line null2. Any background signal for hTLR2 HEK cells was corrected using the parental cell line null1, and any background signal for hTLR2/1 and hTLR2/6 was corrected using the hTLR2KO-TLR1/6 cells.

### Protein precipitation of conditioned media

Sterile 40 mL aliquots of conditioned culture medium was stored at −20°C before thawing at room temperature for use. Proteins were precipitated from culture medium using TCA. To 20 mL of conditioned culture medium, 66% (wt:wt in MQ water) TCA was added to a final concentration of 10% wt:wt with stirring. The sample was kept at 4°C for at least 2 h to encourage precipitation before centrifugation (15,000 × *g*, 15 min). The supernatant was carefully decanted and the pH adjusted before dialysis, 2 mL of 1 M Tris base was added to act as a buffer before dropwise sodium hydroxide was used to obtain a pH between 4 and 7. Sample was added to 3,500 MWCO dialysis tubing (ThermoFisher, Cat. No. 88244), dialyzed extensively against deionized (DI) water (1 L, then a continuous flow for at least 30 h), and dried by lyophilization. Mock TCA precipitated samples were treated identically, except water was added to the sample in place of the 66% TCA solution. Methanol or acetone-based precipitation (95) was employed as orthogonal protein depletion methods. About 20 mL of conditioned culture medium was dialyzed extensively against DI water in 3,500 MWCO dialysis tubing and dried via lyophilization. Typically, 40–70 mg of material was recovered from 20 mL of medium. Each sample was resuspended in 2 mL of MQ water. Sodium chloride (1 M solution in MQ water) was added to the acetone-precipitated sample to a final concentration of 100 mM. Each 2 mL sample was split into “treated” or “mock precipitated,” and 4 mL of methanol (4 eq.) or acetone was added (80% vol:vol organic solvent final concentration) to each treated sample. For mock precipitated samples, MilliQ water was added. Samples were incubated for 2 h at 22°C before centrifugation (15,000 × *g*, 15 min). Sample supernatants were carefully separated, and the organic solvent was removed under reduced pressure. The supernatant samples were diluted to a final volume of 5 mL with addition of MilliQ water, dialyzed extensively against MilliQ water in a 3,500 MWCO dialysis cassette (ThermoFisher, Cat. No. 66110), and dried with lyophilization. The pellet from each protein precipitation, or its mock precipitated control, was resuspended in 2.5 mL of 50 mM Tris (pH 7.5), 100 mM NaCl, and 8 M urea with bath sonication for 1 min every 20 min. After fully dissolving, 2.5 mL of 50 mM Tris (pH 7.5) and 100 mM NaCl were added. Each sample was dialyzed extensively against MQ water in a 3,500 MWCO dialysis cassette, and dried by lyophilization. The mass of the lyophilized was measured and samples were then resuspended in DI water to 5 mg/mL before being back diluted to 1× (accounting for original volume from which they were prepared). Samples diluted to 1× were stored at −20°C until further use in *in vitro* IL-10 assays and TLR2 reporter assays described above.

### Protein digestion with proteinase K and pronase

Before digestion with protease, conditioned culture medium was dialyzed extensively against DI water in 3,500 MWCO dialysis tubing and dried via lyophilization. Typically 40–70 mg of material was recovered from 20 mL of medium. The dried sample was fully resuspended in 2.5 mL of 40 mM Tris (pH 7.45), 4 mM NaCl, 3 mM CaCl_2_, 4 M urea before another 2.5 mL of 40 mM Tris (pH 7.45), 4 mM NaCl, 3 mM CaCl_2_ was added to reduce the urea concentration to 2 M. Samples were digested at 56°C for 18 h using Proteinase K (40 µg/mL final concentration from a 1 mg/mL stock solution prepared in phosphate buffer saline; Fisher BioReagents, Cat. No. BP1700-50) or pronase (100 µg/mL final concentration from a 20 mg/mL stock solution prepared in DI water; Sigma Aldrich, Cat. No. 10165921001). Samples were heat inactivated at 95°C for 15 min, centrifuged to remove insoluble or precipitated protein (15,000 × *g*, 15 min), dialyzed extensively against DI water in 3,500 MWCO dialysis cassette (ThermoFisher, Cat. No. 66110), and dried by lyophilization. Mock digested samples were treated identically except the protease was omitted. The mass of the lyophilized was measured and samples were then resuspended in DI water to 5 mg/mL before being back diluted to 1× (accounting for original volume). Samples diluted to 1× were stored at −20°C until further use in *in vitro* IL-10 assays and TLR2 reporter assays described above. To determine the efficacy of protein depletion, 45 µL of the treated materials and appropriate controls were mixed with 15 µL of Laemmli-BME (beta-mercaptoethanol), heated to 95°C for 5 min, and then 27.5 µL was loaded on two independent SDS-PAGE gels. The gels were then subjected to electrophoresis at 50 V for 5 min, followed by 100 V volts for 75 min. One gel was then silver-stained to assess protein depletion while the other gel was emerald-stained to assess carbohydrate depletion.

### Liquid-liquid extraction of conditioned media

Following extensive dialysis against DI water and drying by lyophilization (see above), the sample for liquid-liquid extraction was resuspended at 10 mg/mL in 5 mL of DI water and added to a separatory funnel. To the sample, 3 mL of chloroform was added, and the funnel was repeatedly inverted to mix. The organic phase and interface were removed. The sample was extracted again by adding 1 mL of chloroform to the remaining aqueous phase. The organic phase and interface were removed and pooled with those from the first extraction. The organic phase and interface were dried with centrifugal evaporation. The aqueous phase was dialyzed extensively against water and dried by lyophilization. The mass of the lyophilized or dried sample was measured and samples were then resuspended in DI water to 5 mg/mL before being back diluted to 1× (accounting for original volume from which they were prepared). Samples diluted to 1× were stored at −20°C until further use in the cell-based assays described above.

### Assessment of *BT4295* expression via a *B. theta*-specific CD4^+^ T cell activation assay

Generation of bone marrow dendritic cells (BMDCs): Bone marrow was isolated from the femur and tibia using a tube-in-tube centrifugation method, where a hole was poked in the bottom of a sterile PCR tube using a sterile 18G needle which was then placed in a sterile Eppendorf tube. The ends of the femur and tibia were removed and the bones were placed open side down in the PCR tube. The tubes were centrifuged briefly to remove the bone marrow and lysed with ACK lysis buffer (100 µL per tube for 3 min at room temperature) and counted as described for splenocytes. Bone marrow cells were resuspended in sterile BMDC media (complete RPMI: RPMI 1640 supplemented with l-glutamine, HEPES (20 mM final concentration; Gibco, Cat #15630-080), 10% vol/vol final concentration heat-inactivated FBS, 100 units/mL Penicillin, and 100 μg/mL Streptomycin supplemented with 0.05 mM 2-mercaptoethanol (Sigma, M3148). The cells were plated at 2 × 10^6^ cells per non-tissue culture-treated petri dish in 10 mL BMDC media. About 20 μL of 10 μg/mL recombinant GM-CSF (20 ng/mL final concentration, Peprotech, Cat #315-03) was added per petri dish to promote differentiation into dendritic cells. The cells were incubated at 37°C with 5% CO_2_ and 95% humidity for 10 days where the media were refreshed every 3 days. On day 3 of culture, 10 mL of BMDC media supplemented with 20 μL of 10 μg/mL recombinant GM-CSF (per petri dish) was added directly on top of the cells. On day 6, half of the media were removed from each petri dish, into a 50 mL conical and the cells were pelleted (454 × *g* for 5 min). The supernatant was removed and the cell pellet was resuspended in 10 mL BMDC media supplemented with 10 μL of 10 μg/mL recombinant GM-CSF (per petri dish). About 10 mL of resuspended cells was added to each petri dish. This same process was repeated on day 9, except cells were resuspended in 10 mL of BMDC media supplemented with 5 μL of 10 µg/mL recombinant GM-CSF (per petri dish). On day 10, differentiated BMDCs were harvested by gently pipetting up and down and the supernatant was collected in a 50 mL conical tube. The cells were pelleted by centrifugation (as above), resuspended in 5 mL BMDC media, and counted with a hemocytometer. BMDCs were plated in a flat bottom 96-well plate at 5 × 10^4^ cells/well in 50 μL. *B*. theta^*Δtdk*^ and *B*. theta^*ΔtdkΔBT4295*^ were grown in TYG overnight to stationary phase. The OD_600_ of each culture was measured. About 1 mL of each culture was placed in a sterile Eppendorf tube and the cells were pelleted by centrifugation (16,000 × *g* for 1 min). The supernatant was removed and the pellet was resuspended in the same volume of sterile PBS. The cells were pelleted again (as above), the supernatant was removed and the pellet was resuspended in sterile PBS. The cells were heat-killed by boiling for 90 mins at 95°C, the OD_600_ was remeasured for each bacterium, the cells were pelleted as above and the pellet was resuspended in a volume of BMDC media to equal the original OD_600_. Dilutions of each heat-killed culture were made such that 50 µL of the bacterial cells were added to the BMDCs and the final OD_600_ of the well was 0.0001, 0.001, 0.01, 0.1, or 1.0. BMDCs and the heat-killed *B. theta* strains were incubated in a total of 100 μL media overnight at 37°C with 5% CO_2_ and 95% humidity.

The following day naïve CD4^+^ T cells were isolated from the spleens of *B. theta*-specific TCR transgenic mice (BθOM mice; [77, 78]). Splenocytes were isolated as described for the *in vitro* splenocyte assay and naïve CD4^+^ T cells were isolated using the Miltenyi Biotec kit (Cat #130–104-453) following the manufacturer’s instructions. The enriched cells were pelleted by centrifugation as described above, resuspended in 1 mL of BMDC media, and counted using a hemocytometer. Isolated naïve CD4^+^ T cells were plated with the BMDC/*B. theta* culture (100 μL of naïve CD4^+^ T cells at 5 × 10^5^ cells/mL). The cells were incubated for 2 days at 37°C with 5% CO_2_ and 95% humidity, following which CD4^+^ T cell activation was assessed using flow cytometry. The cells were collected into 96-well U bottom plates and washed two times with PBS/0.1% BSA, Fc receptors blocked, before being stained for flow cytometry using the following antibodies: anti-CD45.1, anti-TCRvβ12, anti-CD4, anti-CD44, anti-CD62L, CD69, and fixable live/dead dye (see Table S1B for antibody details). Stained cells were washed two times with PBS/0.1% BSA and fixed overnight using a paraformaldehyde-based fixative (BioLegend, Cat. #420801). Fixative was washed off using PBS/0.1% BSA and cells were resuspended in PBS/BSA for acquisition on an LSR Fortessa flow cytometer (BD Biosciences). Data were analyzed using FlowJo software.

### OMV isolation and quantification

OMVs were isolated from conditioned media as previously described (55, 96). Briefly, a set volume of conditioned media was put into 1.5 mL ultracentrifugation tubes (ThermoScientific, Cat. #314352H01) and spun at 100,000 × *g* for 2 h at 4°C. Following the spin, the supernatant was removed and the pellet (containing the OMVs) was resuspended and washed in 1 mL of sterile PBS (all PBS was first double filtered through a 0.1 μm PES filter [Thermo Scientific, Cat. #5670010] before use). This resuspension was spun down again at 100,000 × *g* for 2 h, the supernatant was removed, and pellet containing the washed OMVs was resuspended in 0.1 μm filtered PBS. OMVs were concentrated for Qubit concentration analysis. To quantify using Qubit (Invitrogen, Cat. #Q33211), OMVs were isolated from 1 mL aliquots of conditioned media from *B. theta*^*Δtdk*^ and mutant strains as described above. OMVs from 1 mL of conditioned media were concentrated 50× by resuspending in 20 μL of sterile filtered PBS. The concentration was measured in μg/mL and then the 1× original concentration of OMVs was calculated.

### Statistical analysis

Statistical analyses were performed in GraphPad Prism version 10.0.0. Details of the specific statistical tests used are included in the appropriate figure legends.

## SUPPLEMENTAL MATERIAL

Fig. S1 spectrum.01669-24-s0001.tif HEK293 human TLR2/1 and human TLR2/6 reporter cell line activation with Pam2CSK4 and Pam3CSK4 controls, related to Fig. 1.

Fig. S2 spectrum.01669-24-s0002.tif TCA treatment and protein digestion of *B. theta*VPI-5482 conditioned media does not inhibit splenocyte activation, related to Fig. 2.

Fig. S3 spectrum.01669-24-s0003.tif mTLR2 activation and splenocyte IL-10 induction by TCA, methanol, or acetone/NaCl treated conditioned media or precipitated pellet compared to mock treated controls, related to Fig. 2.

Fig. S4 spectrum.01669-24-s0004.tif Proteinase K and pronase digestion of *B. theta* VPI-5482 conditioned media, related to Fig. 2.

Fig. S5 spectrum.01669-24-s0005.tif Biochemical characterization of immunomodulatory factor produced *B. theta*-VPI 5482.

Fig. S6 spectrum.01669-24-s0006.tif *B. theta* produced immunodominant lipoprotein and glycine lipids are not essential for IL-10 induction.

Fig. S7 spectrum.01669-24-s0007.tif *glsB*, *BT4295*, and *BT1549* mutant and parent strain growth curves, related to Fig. 4 and Fig. S6.

Fig. S8 spectrum.01669-24-s0008.tif Additional biological replicates of delta*BT1549*, delta*glsB*, and delta*BT4295* conditioned media IL-10 induction, related to Fig. 3, Fig. 4, Fig. S6.

Fig. S9 spectrum.01669-24-s0009.tif Prediction of *BT1549* as a lipoprotein by SignalP 6.0 and impact of deletion of BT1549 on outer membrane vesicle biogenesis.

Table S1 spectrum.01669-24-s0010.xlsx Strains, vectors, and primers used in this study, and supplementary figure legends.

ASM does not own the copyrights to Supplemental Material that may be linked to, or accessed through, an article. The authors have granted ASM a non-exclusive, world-wide license to publish the Supplemental Material files. Please contact the corresponding author directly for reuse.
