# Supplementary figures and images for: BT1549 coordinates the in vitro IL-10 inducing activity of Bacteroides thetaiotaomicron
Source: Microbiol Spectr. 2025 Jan 27;13(3):e01669-24. doi: 10.1128/spectrum.01669-24 (PMC11878027; doi:10.1128/spectrum.01669-24)

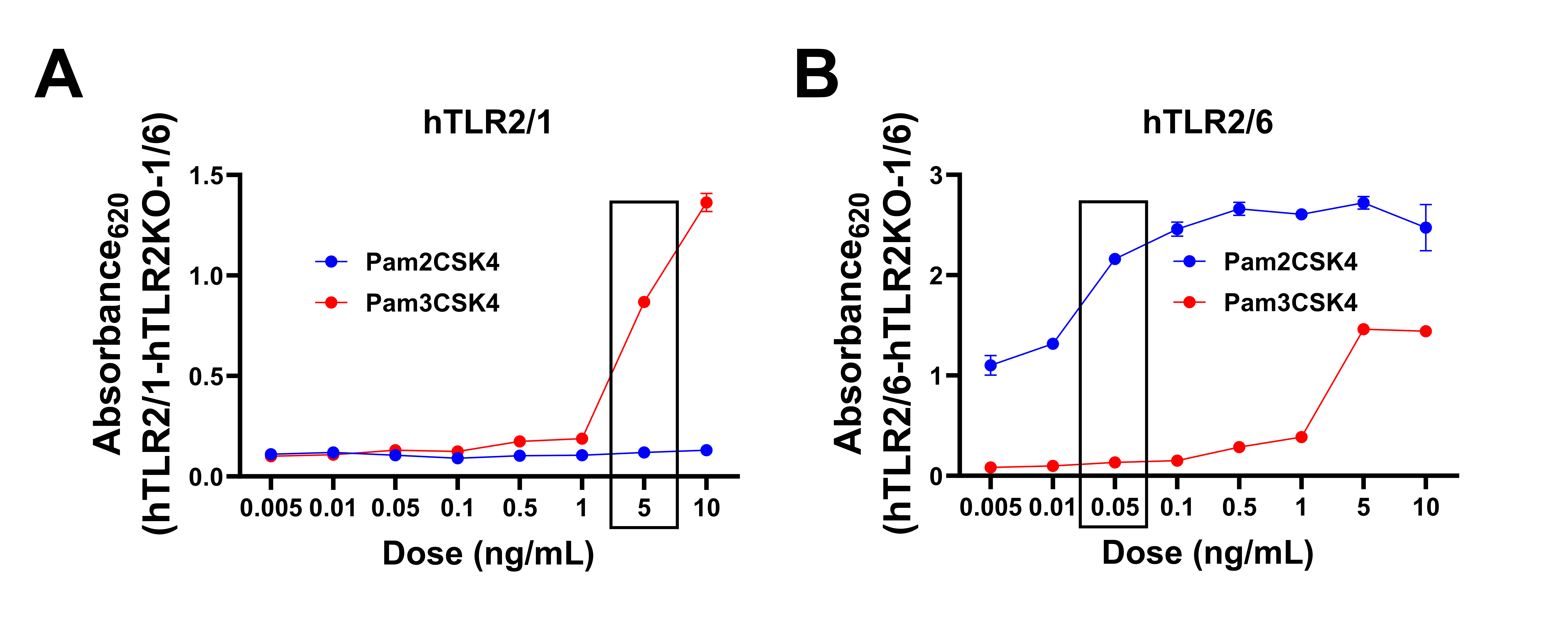

Supplement: Fig. S1 — HEK293 human TLR2/1 and human TLR2/6 reporter cell line activation with Pam2CSK4 and Pam3CSK4 controls, related to Fig. 1. [file spectrum.01669-24-s0001.tif]

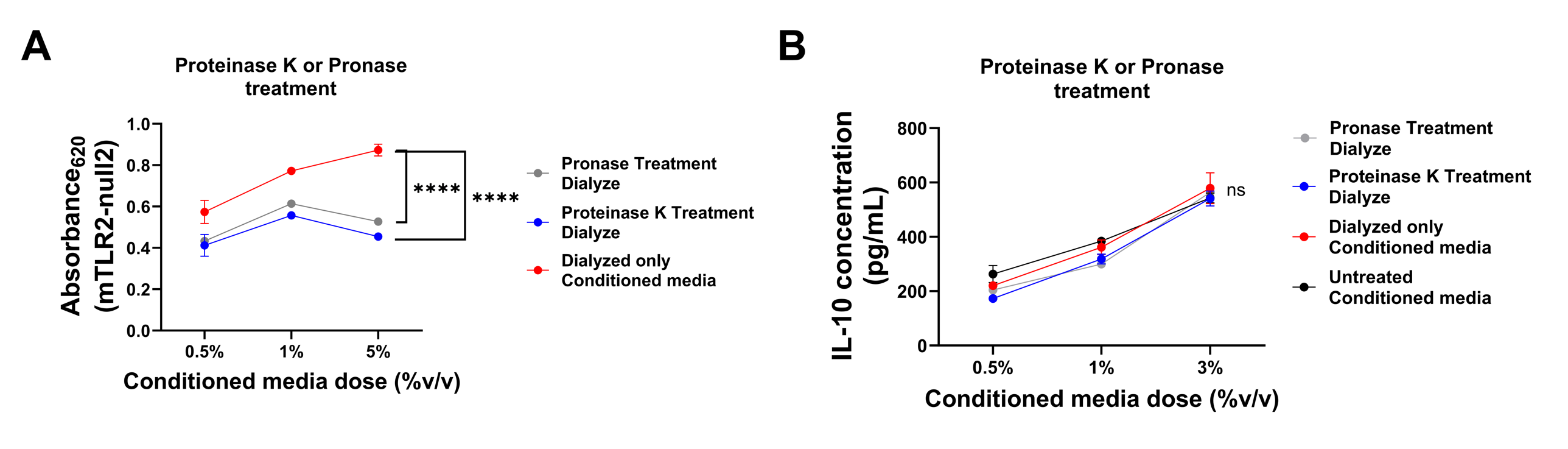

Supplement: Fig. S4 — Proteinase K and pronase digestion of B. theta VPI-5482 conditioned media, related to Fig. 2. [file spectrum.01669-24-s0004.tif]

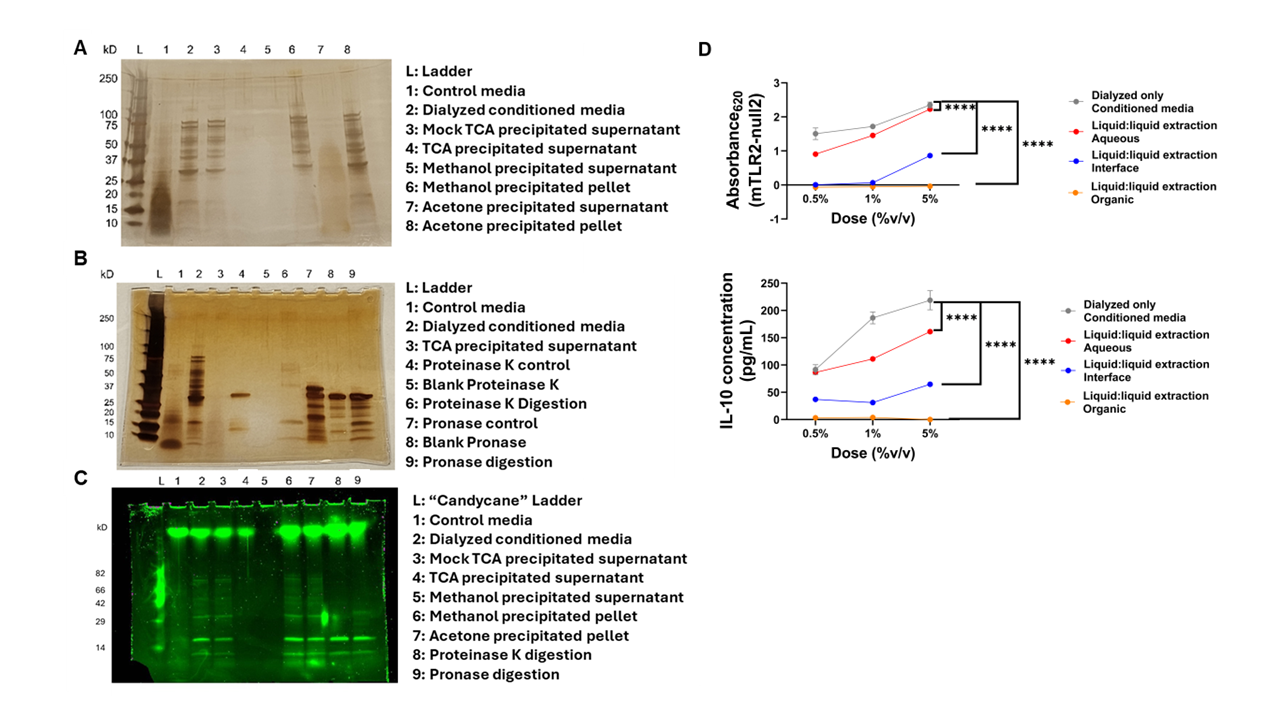

Supplement: Fig. S5 — Biochemical characterization of immunomodulatory factor produced B. theta-VPI 5482. [file spectrum.01669-24-s0005.tif]

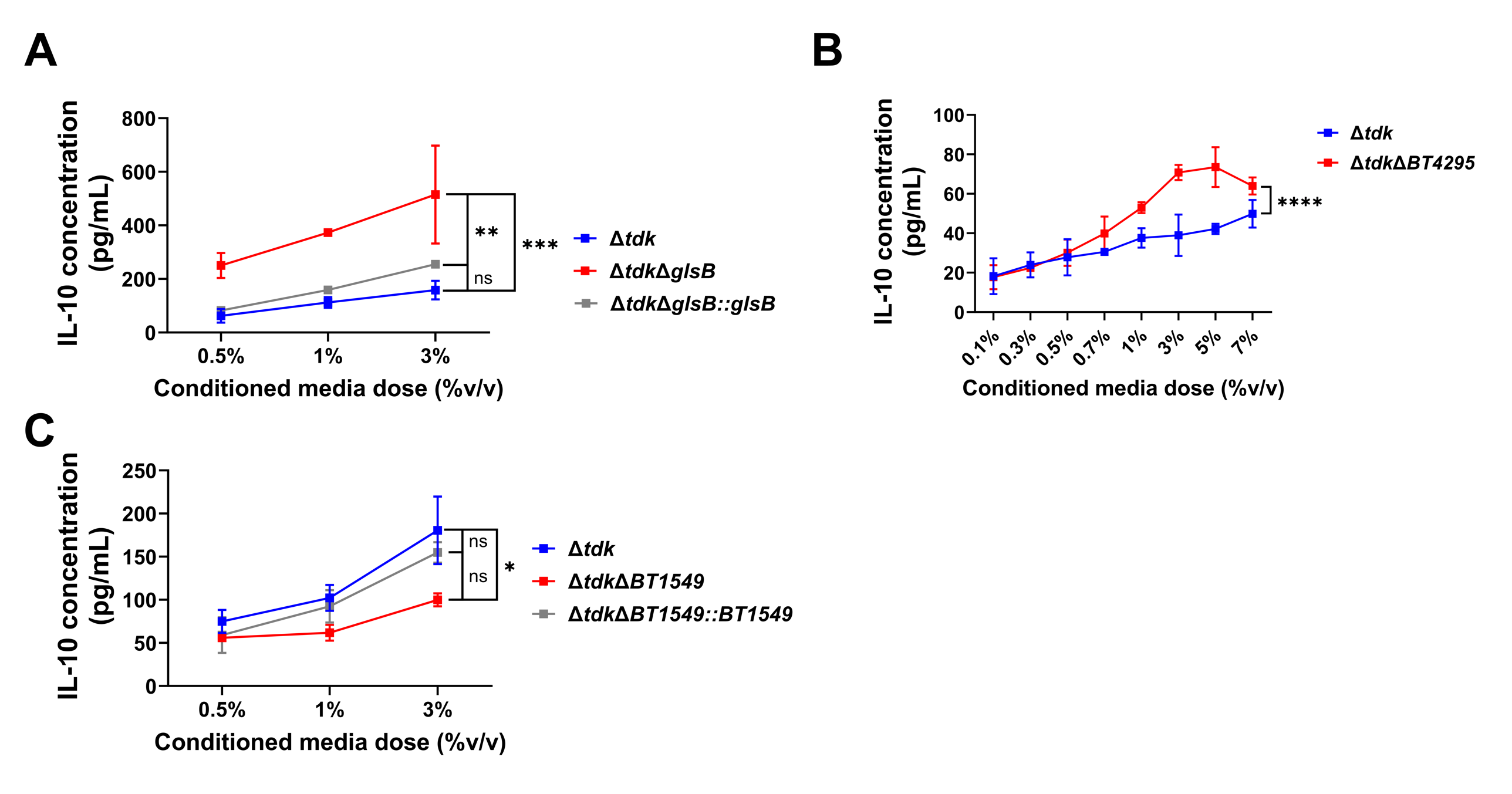

Supplement: Fig. S8 — Additional biological replicates of deltaBT1549, deltaglsB, and deltaBT4295 conditioned media IL-10 induction, related to Fig. 3, Fig. 4, Fig. S6. [file spectrum.01669-24-s0008.tif]

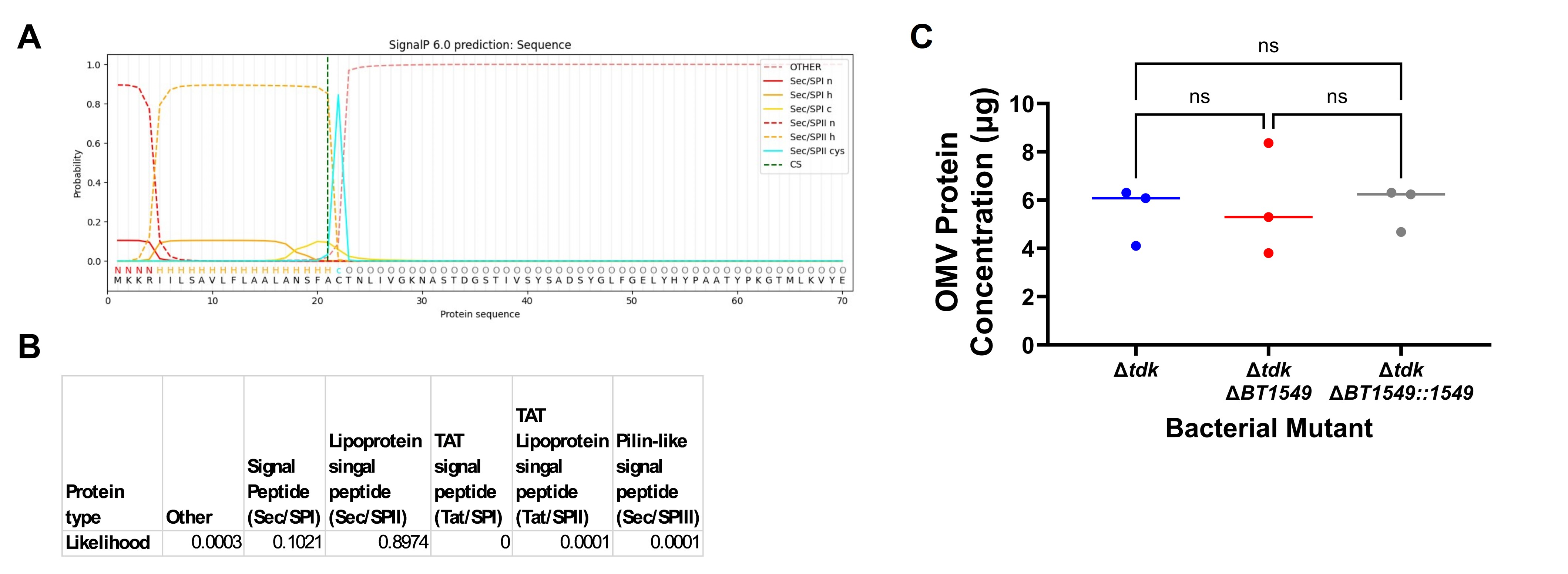

Supplement: Fig. S9 — Prediction of BT1549 as a lipoprotein by SignalP 6.0 and impact of deletion of BT1549 on outer membrane vesicle biogenesis. [file spectrum.01669-24-s0009.tif]
